# Management of scope-induced type I duodenal perforations: Over-the-scope clip versus surgery

**DOI:** 10.1007/s12664-021-01152-0

**Published:** 2021-05-11

**Authors:** Amol S. Dahale, Siddharth Srivastava, Sundeep Singh Saluja, Sanjeev Sachdeva, Ashok Dalal, Shivakumar Varakanahalli

**Affiliations:** 1 Department of Gastroenterology, Dr. D Y Patil Medical College and Hospital, Pimpri, Pune, 411 018 India; 2 Department of Gastroenterology, G B Pant Institute of Postgraduate Medical Education and Research, 1, J L N Marg, New Delhi, 110 002 India; 3 Department of Gastrointestinal Surgery, G B Pant Institute of Postgraduate Medical Education and Research, New Delhi, 110 002 India

**Keywords:** Adverse effects, Antibiotics, Endoscopy, Endoscopic retrograde cholangiopancreatography, Endoscopic ultrasonography, Hospital stay, Intestinal perforation, Outcome, Pneumoperitoneum

## Abstract

**Background:**

Scope-induced duodenal perforation is a life-threatening complication and surgery remains the standard of care. With the advent of over-the-scope clip (OTSC), scope-induced perforations are increasingly managed conservatively, though there is no study comparing this form of non-surgical treatment with surgery. We aimed to compare OTSC and surgery in the management of scope-induced perforation of the duodenum.

**Methods:**

We retrospectively collected data of scope-induced duodenal perforation patients. Perforations identified and treated within 24 h of procedure were analyzed. Factors analyzed were spectrum, etiology, baseline parameters, perforation size, outcome, comorbidities, and duration of hospital stay.

**Results:**

A total of 25 patients had type I duodenal perforations, out of whom five were excluded due to delayed diagnosis and treatment. Of the twenty, eight were treated with OTSC placement while the rest underwent surgery. Age was comparable and the majority were females. Baseline parameters and comorbidities were similar in both the groups. The median size of perforation was 1.5 cm in both the OTSC group and the surgical group. All patients were treated with standard of care according to institutional protocols. Patients in the OTSC group were started orally after 48 h of OTSC placement, while in the surgery group median time to oral intake was 7 days. Two patients in the surgical group died while there was no mortality in the OTSC group (*p* = 0.48). Median hospital stay was shorter in the OTSC group (2 days vs. 22 days, *p* = 0.003).

**Conclusions:**

OTSC is a feasible and better option in type I duodenal perforations with a shorter hospital stay.

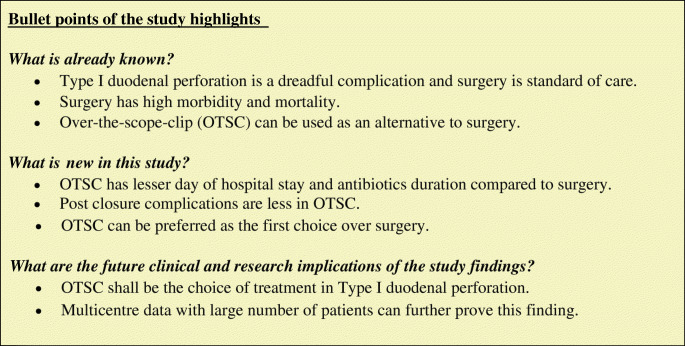

## Introduction

Endoscope-induced perforation is a rare but lethal complication of diagnostic and therapeutic gastrointestinal (GI) endoscopy [1–3]. The data are scarce due to the rarity of the occurrence and underreporting of complications. Scope-induced perforation is generally full thickness defect in the GI wall and traditionally has been treated surgically. A side-viewing endoscope used for endoscopic retrograde cholangiopancreatography (ERCP) and echo-endoscope used for endoscopic ultrasound (EUS) are more notorious for perforations due to their large size, side and oblique viewing images, and stiff distal ends. Duodenum is the most common site of injury with side-viewing scope. Stapfer et al. classified such injuries as type I and surgery has been the primary modality of treatment considering their grievous nature [2]. Surgery is definitely associated with morbidity and substantial mortality (up to 46.2%) [4]. Metallic clip has been in use for hemostatic purpose since long time, but their use in GI defects is recent one [5]. Later metallic clips were being increasingly used for closure of GI defects during endoscopic mucosal resection (EMR) and endoscopic submucosal dissection (ESD) with encouraging outcomes [6, 7]. With recent innovation of the over-the-scope clip (OTSC) by a German group (Ovesco Endoscopy AG, Tubingen, Germany), OTSC nowadays is being used more commonly [8]. Another new innovative padlock clip is also on the horizon and can be used with similar effect without occupying endoscopy channel [9]. To date, the data on the OTSC for scope-induced perforation are scarce. First report on OTSC was from a colonoscope-related perforation by Kirschniak et al. [8]. Later porcine studies were done for colon, stomach, and duodenal perforations, respectively, which found encouraging results. It led to widespread acceptance and use of the OTSC for post-EMR or post-ESD GI perforations and post-surgical GI defects and leaks [10–12]. Type I duodenal perforation management data are mostly from the surgical case series [13]. Endoscopic management of type I perforation with the OTSC is limited to case reports and series only [14–21]. Though this preliminary data is encouraging, to date there is no comparative study with surgery, which is the standard of care for type I perforations. We aimed to compare outcome of duodenal perforations treated with either of the modalities, i.e. surgery or OTSC.

## Methods

This is a retrospective study carried out at G B Pant Institute of Postgraduate Medical Education and Research (GIPMER), New Delhi, India, from January 2008 to April 2019. Data on scope-induced perforations were retrieved from endoscopic registers and case sheets of admitted patients. Institutional ethical committee provided waiver for this study as it was retrospective.

### Patients

 The data on clinical spectrum of the patients, primary diagnosis, site of perforation, and size of perforation were noted. Mode of treatment was noted as surgical or endoscopic.

### Inclusion criteria

All adult patients with scope-induced perforation in duodenum were included.

Diagnosis of perforation was confirmed on endoscopy as full thickness defect or on computed tomography (CT) scan of the abdomen showing active contrast leak or intra-operatively by surgeon.

### Exclusion criteria

Patients were excluded from the study if any one or more of the following was present:

1. Age below 18 years
2. Diagnosed after 24 h of endoscopic procedure
3. Treatment started after 24 h of endoscopic procedure
4. Conservative management.

### Treatment protocol

All patients were treated by the team of gastroenterologist, GI surgeon, and a dedicated radiologist with institutional protocol for management of iatrogenic perforations. The protocol included keeping patient nil by mouth and Ryle’s tube insertion with continuous drainage to divert gastric contents. Intravenous (IV) antibiotics were given to all (imipenem, ofloxacin, and metronidazole for broad spectrum coverage followed by change as per sensitivity pattern). Abdominal roentgenogram was done in all patients. CT scan with oral contrast was done in patients in whom the  diagnosis was in doubt. If endoscopic view of full thickness defect or documentation of air under the diaphragm or active leak of contrast on CT was seen, patients were taken for definitive treatment without waiting for abdominal signs to develop. Informed consent was taken from patients for further definitive treatment either by surgery or OTSC.

### Surgical management

Surgery done was primary duodenal repair with pyloric exclusion, with gastrojejunostomy, feeding jejunostomy (FJ), and drain placement in peritoneal cavity. Additional surgical procedure for underlying disease was done if the situation demanded and was feasible. FJ feed was started within 48 h while oral feeds were started as per judgement of the treating clinician. Intravenous antibiotics were continued until removal of drain. Patients were discharged after removal of drain.

### Endoscopic management

For the OTSC patients, size and site of perforation were identified. Size of perforation was determined by measuring against opened biopsy forceps (open cup size of 5 mm). OTSC clip size 12/6 or 11/6 (Ovesco, Tübingen, Germany) was used for closure of defect. Post-OTSC placement, patients were kept nil by mouth overnight followed by oral contrast study. If no leak was demonstrated, then oral feed was started. After 24 h of the well-tolerated oral feed, patients were discharged with advice to follow-up weekly as outpatient for 2 weeks, then monthly.

Decision to treat with surgery or OTSC was subject to availability of the latter and was taken by team consisting of gastroenterologist, GI surgeon, and radiologist. Most patients before 2014 were treated with surgery; later, with the availability of the OTSC, most of the patients were treated with the OTSC.

All patients were followed up for 6 months. Duration of hospital stay and cause of death were compared in both the groups.

### OTSC placement

After confirmation of perforation, patient was taken for the OTSC placement. All procedures were done using a therapeutic forward viewing endoscope TJF-C180V, (Olympus Inc., Tokyo, Japan). Procedure was done with patient in left lateral decubitus position in conscious sedation. Endoscope with the clip mounted on distal tip was advanced to the site of perforation. Endoscope was positioned so as to bring perforation site in center of vision. After this, suction was applied until both the ends of perforation were inside the clip applicator. Immediately after this, with suction continuing, clip was applied with same maneuver as endocopic variceal bands are applied. Post-deployment, the clip was inspected for position and residual perforation, if any. We did not use an anchor or twin grasper in any patient as we were successful with the suction method in all the patients.

### Statistical analysis

Mean and median were used for continuous data expression. Continuous parametric data were compared using independent Student *t* test. Continuous nonparametric data were compared using the Mann-Whitney *U* test. Categorical variables were compared using Fischer’s exact test. *P*-value < 0.05 was considered significant. All statistical analyses were done using Statistical Products and Services Solution (SPSS) software version 20.0 (SPSS, Chicago, IL, USA).

## Results

**Fig. 1:**
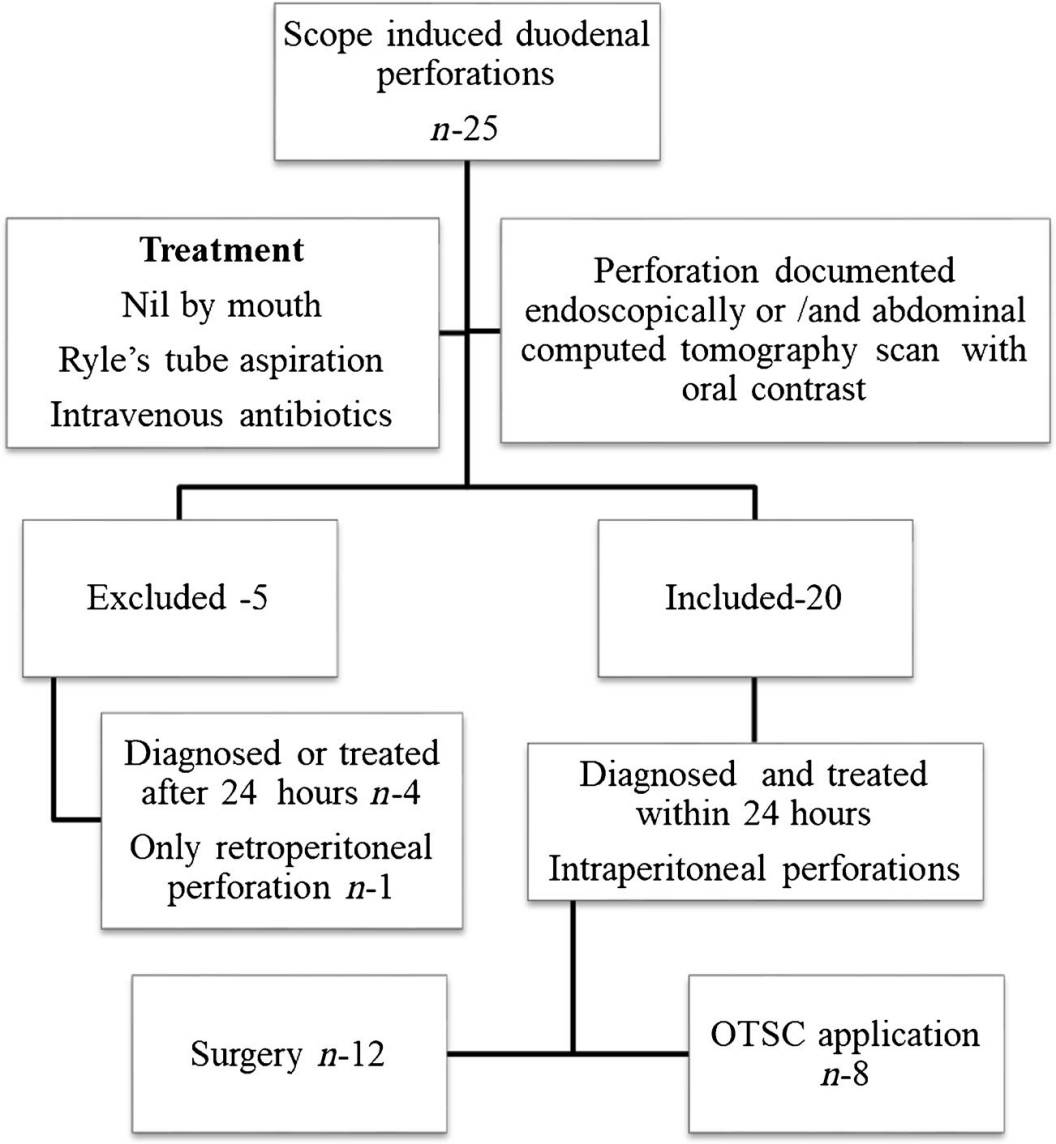
Patients with type I perforations analyzed for study. *OTSC *Over-the-scope clip

During the study period, 26,257 therapeutic ERCP and 5987 EUS were done. Twenty-seven (0.08%) had scope-related perforations of whom 25 (0.07%) had duodenal perforations and the rest two had stomach and gastroesophageal junction perforation. Of duodenal perforations, one patient had only retroperitoneal perforation and was treated conservatively, who was excluded from the analysis. Of the remaining four, two had delayed diagnosis and two had delayed treatment, all of whom were excluded from the analysis. Total 20 patients were enrolled for analysis in this study. Of them, eight underwent OTSC as definitive treatment while 12 underwent surgery (Fig. 1).

### OTSC group

**Fig. 2:**
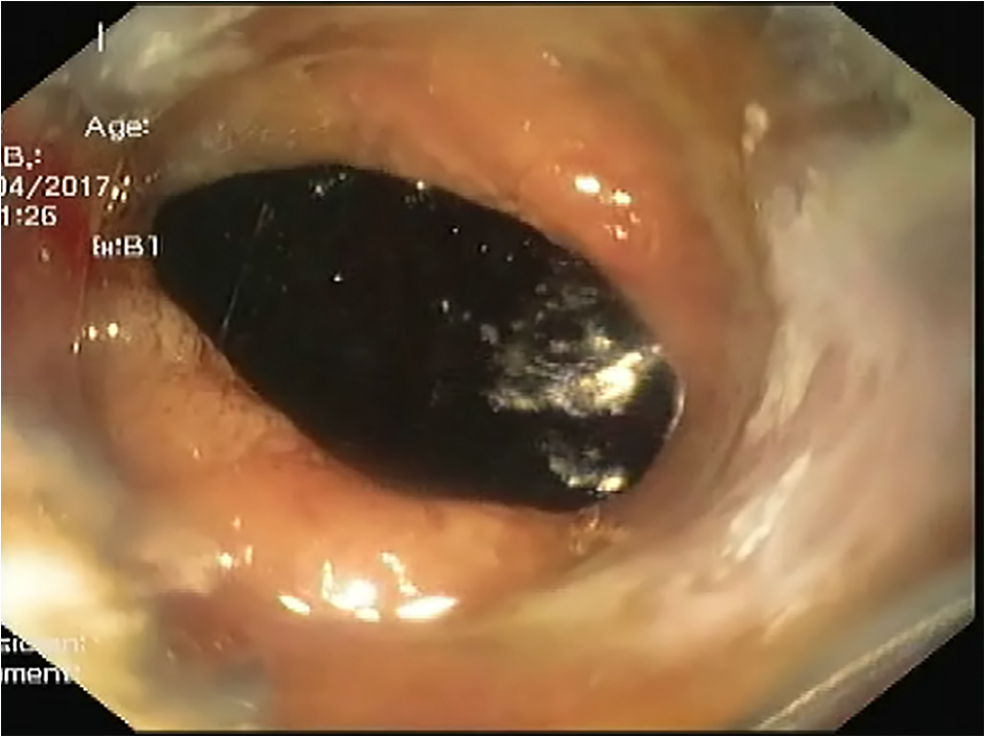
Lateral duodenal wall perforation

**Fig. 3:**
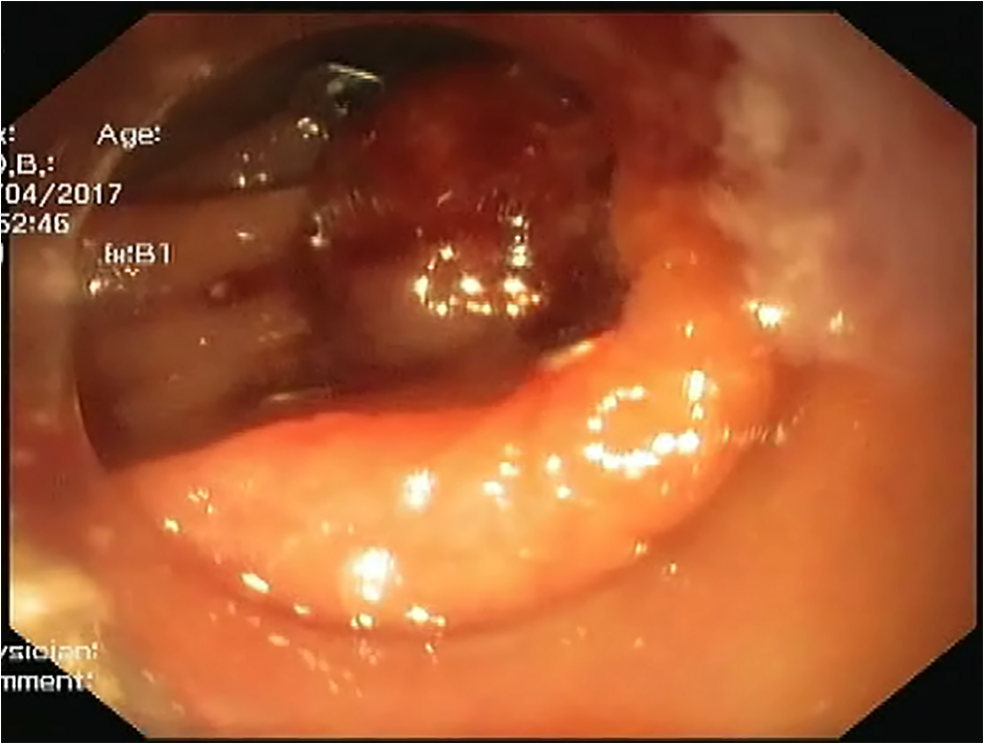
Same perforation after  over-the-scope clip application

OTSC was applied in the eight patients of type I duodenal perforation (Figs. 2 and 3). Median age was 65 years (range 45–67 years) and seven of them were females. Four patients had carcinoma gallbladder and one had carcinoma head of pancreas, while the other three had gallstone with choledocholithiasis. Four patients with the malignant etiology had evidence of metastasis and two had diabetes mellitus and hypertension as comorbidities. Two perforations occurred during EUS while in six during ERCP. Four patients had perforation in D1 and D2 junction extending into D2 along the posterolateral wall while the remaining four had it on the lateral wall of the second part of the duodenum. Median size of perforation was 1.5 cm (range 1.4–2 cm). All patients were detected during endoscopy itself and were treated within 12 h of detection. Only one OTSC for each  perforation was needed. No additional closure devices or clip of any type was used. Suction method was used for approximation of edges. Closure was confirmed endoscopically on table. After closure, the patients were kept nil by mouth overnight followed by an oral Gastrografin study to document closure and clip position. Additional CT with oral contrast was done for the initial four patients to ensure success of procedure and to look for any collection. Later, we reserved it only for those having suspected collection, pain, or fever. Once closure was confirmed, all patients were allowed orally on the second day and discharged after 24 h. IV antibiotics were given during hospitalization followed by oral antibiotics for 2 weeks during discharge and the patients were followed weekly for an initial 1 month, then monthly for the next 2 months. Median stay of patients was 2 days (range 2–3 days). There was  no complications related to OTSC. There was no mortality within 3 months. At 6 months, two patients died due to underlying metastatic disease unrelated to perforation event.

### Surgery group

**Fig. 4:**
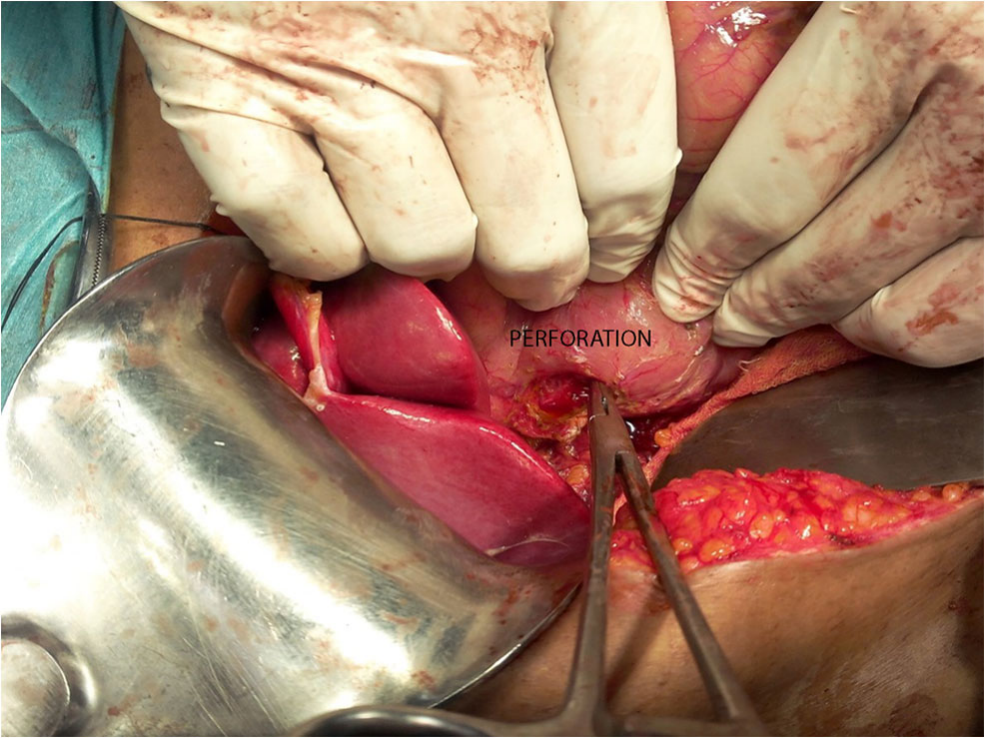
Lateral duodenal wall perforation at surgery

**Fig. 5:**
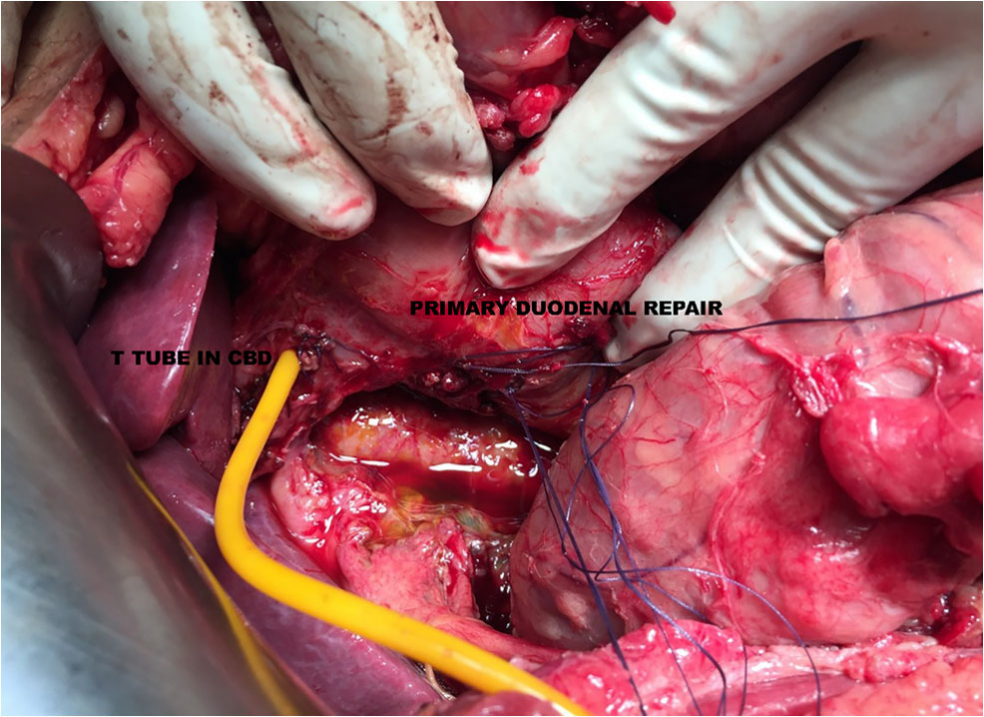
Primary duodenal repair is being done in one patient

Surgery was done in twelve patients for type I duodenal perforation. Four patients had carcinoma gallbladder, one had periampullary carcinoma, and seven had gallstone disease with choledocholithiasis. Median age was 57 years (range 40–75 years) with 9 females. All patients had perforation in the duodenum (Fig. 4) and all perforations occurred during ERCP with a side viewing endoscope. Four had perforation at D1 and D2 junctions extending into D2, while others had perforation in D2. All perforations were on the posterolateral wall. Median size of perforation was 1.5 cm (range 1–3 cm). All patients were operated within 24 h of perforation of whom 11 were operated within 12 h. Surgery undertaken was primary duodenal repair (Fig. 5) with pyloric exclusion, with gastrojejunostomy, FJ, and drain placement. The patients with cholelithiasis with choledocholithiasis also underwent cholecystectomy and common bile duct exploration with stone removal and T tube placement. Four patients with carcinoma gallbladder were unresectable and underwent additional tube biliary drainage. One patient with periampullary carcinoma also underwent cholecystojejunostomy. FJ feed was started for all the patients on the second or third day. Oral feeds were started after 5 days; the decision to start oral feed was subject to patient’s condition and healing of perforation. Median time to start oral feed was 6.5 days (range 2–25 days). The median duration of antibiotics given was19.5 days (range 7–45 days). The patients were discharged once the drain was taken out and the patients were tolerating orally well. Later, FJ was removed on outpatient basis after 3 to 4 weeks. The patients in the surgical group had a median hospital stay of 22.5 days (range 9–51 days). Two patients did not survive (16%). Cause of death in both was sepsis and multiorgan failure. One died on postoperative day 30 while the other succumbed on the 37th postoperative day. There was one or more complications in seven patients. Two patients had leak from the wound site and were managed conservatively. Two had delirium and had spontaneous recovery within 2 days, one of whom also had left lower foot gangrene as thromboembolic phenomenon. One patient had local wound site infection, which was treated with wound care and antibiotics. Two patients had sepsis with multiple organ failure, one whom also had type 1 respiratory failure while another had persistent leak from the wound site, both of whom did  not survive.

### Comparison (Table 1)

Baseline blood parameters, etiology, and comorbidities were comparable in both groups. Size of perforation was slightly larger in the surgical group but statistically insignificant. Hospital stay was significantly shorter for the OTSC group (2.4 days vs. 24 days, *p*-value 0.001). Surgical group received IV antibiotics for more duration (2 days vs. 20 days, *p*-value 0.001). There was no mortality in the OTSC group compared to 16% in the surgical group, but this difference was not statistically significant (*p*-value 0.52).

**Table 1:** Demographic, clinical, and outcome parameters of the over-the-scope clip (OTSC) and surgical group

Parameter	OTSC group (*n* = 8)	Surgery group (*n* = 12)	*p*-value
Age (years)	65 (45–67)	57 (40–75)	0.77
Sex (male:female)	1:7	1:11	0.25
Hemoglobin (gm/dL)	11.20	11.90	0.66
Total leukocyte count (cells/μL)	9800	10550	0.60
Bilirubin (mg/dL)	1.2	0.75	0.10
Albumin (gm/dL)	2.90	3.05	0.53
Etiology (benign:malignant)	3:5 (40%:60%)	7:5 (60%:40%)	0.35
Comorbidities present	4 (50%)	4 (33%)	0.63
Size of perforation (cm)	1.5 (1.4–2.0)	1.5 (1–3)	0.86
Duration of intravenous antibiotics in days	2 (2–3)	19.50 (7–45)	0.001
Oral feed started on day	2	6.5 (2–25)	0.013
Post-procedure complications	0	7 (58%)	0.02
Mortality	0	2 (16%)	0.48
Hospital stay (days)	2 (2–3)	22.5 (9–51)	<0.005

Values in bracket are percentage and range wherever mentioned

## Discussion

To the best of our knowledge, this is the largest and first study comparing surgical management with OTSC for type I duodenal perforations. Although this is a retrospective study, uniformity in management protocol makes it robust.  At our center, depending upon the availability of OTSC and if type I perforation is detected on the table, we prefer to manage the same with OTSC. OTSC has been available in India since 2014, so all the patients undergoing OTSC placement had it after 2014; while in the surgical group, seven cases were from before the year 2014 and five were after the year 2014.

Prevalence of all types of perforation during ERCP was 0.6%, while that during EUS reported was 0.09% [22, 23]. Type I duodenal perforations are rarer and have a frequency of 0.12%; in our study, the figure is 0.07% [24]. Type I perforation, which is the scope-induced perforation, is a full thickness defect in the wall of the duodenum, mostly on the lateral wall. It is an unexpected emergency and is fatal, if not managed promptly. Surgical management has been the standard of care for all scope-induced perforations. Even with the standard surgical management, morbidity and mortality are quite high as reported in the literature, varying from 10% to 46.2% [13]. In addition, a patient might require multiple drainage tubes, local wound management, and prolonged hospital stay (average stay 10 to 60 days) [13]. In the present study, mortality was 20% while hospital stay was 22.5 days in the surgical group.

To date, enough data are available for use of the metallic clips in post-EMR and post-ESD GI defects [7]. Development of the OTSC has made larger defects to be covered by use of single or multiple clips. Animal studies have established efficacy of the OTSC in esophageal, gastric, and duodenal full thickness perforations [10–12]. But data on real-world use of OTSC in scope-induced type I perforation in patients are scarce and published only as case reports and case series. To date, only eight reports comprising of 17 patients were published. Seven of these occurred during EUS, six during ERCP, and four during resection of duodenal adenoma [14–21]. The clips were applied with 100% technical and clinical success rate but three patients needed additional closure methods like endoclips, band ligation, or stenting. Similarly, in our study, we had 100% technical and clinical success.

Type I perforation is a big stress on normal physiology. Surgery adds to this stress causing more chances of postoperative complications including delayed healing. In our study, the OTSC was superior to surgery with shorter hospital stay and shorter duration of intravenous antibiotics. Surgery in perforation is also complicated by associated comorbidities. All our patients in the surgical group had drains postoperatively and required prolonged hospital stay. Surgical patients also required prolonged antibiotic compared to the OTSC group. In the surgical group, postoperative complications further increase morbidity of the patients. In our study, post-surgical complications were seen in seven patients (58%) compared to none in the OTSC group. Also, the OTSC patients had been started oral feeds much earlier leading to earlier return to physiological process with no or minimal depletion of nutritional reserves. Though mortality was higher (16%) in the surgery group, this finding did not reach significance mostly due to small number of patients.

Despite this, there are few advantages of offering surgery as the primary mode of treatment like additional definitive surgical procedures for primary diagnosis. But this is not routine and decision is largely based on underlying etiology, general condition of patient, and urgency of procedure. Considering morbidity and mortality of surgery, it appears better to go for definitive procedure later once perforation is tackled by the OTSC, unless it is an emergency. Although there is a report of perforation size of 40 mm being closed using OTSC, defects more than 20 mm in size are unlikely to be tackled by OTSC effectively, so surgery is definitely on cards for such size perforations [20].

Large numbers of our patients were having underlying malignant disease. Considering the underlying nature of the disease, OTSC offers a better option in this group of patients, which cuts down the time to start cancer-directed treatment.

OTSC had a technical and clinical success rate of 100%. However, the success rate depends on the time of the procedure after perforation. In acute perforations, edges are viable and non-necrotic; also, there is no collection and infectious component, which increase clinical success rate. Thus, their use is justified in an acute setting, rather than in chronic setting, in which success rate is relatively low [25]. Size covered by OTSC in our patients ranged from 1.4 to a maximum of 2 cm. A study had already proved OTSC can be used in defects of size 5–20 mm in the stomach and up to 30 mm in the colon. Even defects larger than these can be closed in the colon with the use of multiple clips [11]. After OTSC placement, closure can be confirmed with an oral contrast study, either oral Gastrografin or CT abdomen with oral contrast. CT may be more useful where delayed closure was done or in symptomatic patient in whom a collection may be expected.

Available comparative data on OTSC for the duodenal perforation is only from the pig models [12]. Though the European Society of Gastrointestinal Endoscopy (ESGE) recommends attempt of endoscopic closure of type I duodenal perforations, evidence is weak and comes from small number of patients as mentioned earlier [3]. In this scenario, our study being the only comparative study gains importance.

There are a few limitations to our study. Our study is a single-center, non-randomized, retrospective study and numbers of patients included are small. Apart from this, only those patients who were treated in an acute setting (within 24 h) were included for this study, and the others were excluded. Since surgery is the standard treatment recommended for late detection of leaks (beyond 24 h), we excluded these patients to avoid ambiguity [3, 26]. Though both groups are comparable on most parameters, addition of definitive surgical procedures makes the data heterogeneous. Prospective randomized trials comparing the OTSC vs. surgery are unlikely to occur in the future due to rarity of condition, ethical issues, and difficulty in creating an ideal situation in complex clinical scenarios. Also, our center is a high volume teaching and training university hospital with good expertise in endoscopy; extrapolation of the results of this study to routine clinical practice shall be difficult. Nevertheless, this study gives a proof of concept that type I perforations can be safely and effectively managed without surgery at centers with expertise.

In conclusion, OTSC is not inferior to surgery for scope-induced perforation in terms of hospital stay and duration of antibiotics. OTSC may be offered as a first-line therapy in scope-induced perforation.

## References

[1] Habr-Gama A, Waye JD (1989). Complications and hazards of gastrointestinal endoscopy. World J Surg.

[2] Stapfer M, Selby RR, Stain SC, et al. Management of duodenal perforation after endoscopic retrograde cholangiopancreatography and sphincterotomy. Ann Surg. 2000;232:191–8. 10.1097/00000658-200008000-00007 PMC1421129 10903596

[3] Paspatis GA, Dumonceau JM, Barthet M, et al. Diagnosis and management of iatrogenic endoscopic perforations: European Society of Gastrointestinal Endoscopy (ESGE) Position Statement. Endoscopy. 2014;46:693–711. 10.1055/s-0034-1377531 25046348

[4] Ercan M, Bostanci EB, Dalgic T, et al. Surgical outcome of patients with perforation after endoscopic retrograde cholangiopancreatography. J Laparoendosc Adv Surg Tech A. 2012;22:371–7. 10.1089/lap.2011.0392 22288879

[5] Singhal S, Changela K, Papafragkakis H, Anand S, Krishnaiah M, Duddempudi S (2013). Over the scope clip: technique and expanding clinical applications. J Clin Gastroenterol.

[6] Tsunada S, Ogata S, Ohyama T, et al. Endoscopic closure of perforations caused by EMR in the stomach by application of metallic clips. Gastrointest Endosc. 2003;57:948–51. 10.1016/s0016-5107(03)70051-0 12776053

[7] Minami S, Gotoda T, Ono H, Oda I, Hamanaka H (2006). Complete endoscopic closure of gastric perforation induced by endoscopic resection of early gastric cancer using endoclips can prevent surgery (with video). Gastrointest Endosc.

[8] Kirschniak A, Kratt T, Stüker D, Braun A, Schurr MO, Königsrainer A (2007). A new endoscopic over-the-scope clip system for treatment of lesions and bleeding in the GI tract: first clinical experiences. Gastrointest Endosc.

[9] Anderloni A, Bianchetti M, Mangiavillano B, et al. Successful endoscopic closure of iatrogenic duodenal perforation with the new Padlock Clip. Endoscopy. 2017;49:E58–9. 10.1055/s-0042-124177 28135728

[10] Schurr MO, Hartmann C, Ho CN, Fleisch C, Kirschniak A (2008). An over-the-scope clip (OTSC) system for closure of iatrogenic colon perforations: results of an experimental survival study in pigs. Endoscopy..

[11] Matthes K, Jung Y, Kato M, Gromski MA, Chuttani R (2011). Efficacy of full-thickness GI perforation closure with a novel over-the-scope clip application device: an animal study. Gastrointest Endosc.

[12] von Renteln D, Rudolph HU, Schmidt A, Vassiliou MC, Caca K (2010). Endoscopic closure of duodenal perforations by using an over-the-scope clip: a randomized, controlled porcine study. Gastrointest Endosc.

[13] Turner RC, Steffen CM, Boyd P (2014). Endoscopic duodenal perforation: surgical strategies in a regional centre. World J Emerg Surg.

[14] Voermans RP, Le Moine O, von Renteln D (2012). Efficacy of endoscopic closure of acute perforations of the gastrointestinal tract. Clin Gastroenterol Hepatol.

[15] Doğan ÜB, Keskın MB, Söker G, Akın MS, Yalaki S (2013). Endoscopic closure of an endoscope-related duodenal perforation using the over-the-scope clip. Turk J Gastroenterol.

[16] Gubler C, Bauerfeind P (2012). Endoscopic closure of iatrogenic gastrointestinal tract perforations with the over-the-scope clip. Digestion..

[17] Hadj Amor WB, Bonin EA, Vitton V, Desjeux A, Grimaud JC, Barthet M. Successful endoscopic management of large upper gastrointestinal perforations following EMR using over-the-scope clipping combined with stenting. Endoscopy. 2012;44 Suppl 2 UCTN:E277–8. 10.1055/s-0032-1309861 22933253

[18] Nishiyama N, Mori H, Kobara H, et al. Efficacy and safety of over-the-scope clip: including complications after endoscopic submucosal dissection. World J Gastroenterol. 2013;19:2752–60. 10.3748/wjg.v19.i18.2752 PMC3653149 23687412

[19] Mangiavillano B, Arena M, Morandi E, Santoro T, Masci E. Successful closure of an endoscopic ultrasound-induced duodenal perforation using an over-the-scope-clip. Endoscopy. 2014;46 Suppl 1 UCTN:E206–7. 10.1055/s-0034-1365389 24756298

[20] Angsuwatcharakon P, Prueksapanich P, Kongkam P, Rattanachu-Ek T, Sottisuporn J, Rerknimitr R. Efficacy of the Ovesco Clip for Closure of Endoscope Related Perforations. Diagn Ther Endosc. 2016;2016:9371878. 10.1155/2016/9371878 PMC4884865 27293368

[21] Salord S, Gornals JB, Maisterra S, Pons C, Busquets J, Fabregat J (2012). Endoscopic closure of duodenal perforation with an over-the-scope clip during endoscopic ultrasound-guided cholangiopancreatography. Rev Esp Enferm Dig.

[22] Andriulli A, Loperfido S, Napolitano G, et al. Incidence rates of post-ERCP complications: a systematic survey of prospective studies. Am J Gastroenterol. 2007;102:1781–8. 10.1111/j.1572-0241.2007.01279.x 17509029

[23] Carrara S, Arcidiacono PG, Mezzi G, Petrone MC, Boemo C, Testoni PA. Pancreatic endoscopic ultrasound-guided fine needle aspiration: complication rate and clinical course in a single centre. Dig Liver Dis. 2010;42:520–3. 10.1016/j.dld.2009.10.002 19955025

[24] Cirocchi R, Kelly MD, Griffiths EA, et al. A systematic review of the management and outcome of ERCP related duodenal perforations using a standardized classification system. Surgeon. 2017;15:379–87. 10.1016/j.surge.2017.05.004 28619547

[25] Haito-Chavez Y, Law JK, Kratt T, et al. International multicenter experience with an over-the-scope clipping device for endoscopic management of GI defects (with video). Gastrointest Endosc. 2014;80:610–22. 10.1016/j.gie.2014.03.049 24908191

[26] Avgerinos DV, Llaguna OH, Lo AY, Voli J, Leitman IM (2009). Management of endoscopic retrograde cholangiopancreatography: related duodenal perforations. Surg Endosc.

